# Landscape Structure and Breeding Site Conditions Shape the Urban Distribution of the Two Frog Species, *Dryophytes japonica* and *Rhacophorus schlegelii*

**DOI:** 10.1002/ece3.72845

**Published:** 2026-01-09

**Authors:** Takeshi Osawa, Nozomu Sato, Hiroto Nagaoka

**Affiliations:** ^1^ Graduate School of Urban Environmental Sciences Tokyo Metropolitan University Tokyo Japan

**Keywords:** breeding site, *Dryophytes leopardus*, habitat connection, *Hyla japonica*, paddy field, urbanization

## Abstract

Urbanization poses significant threats to amphibians through habitat loss, fragmentation, and degradation of breeding sites. This study investigated the distribution of two frog species, *Dryophytes japonica* and *Rhacophorus schlegelii* , which have relatively similar ecological traits in paddy fields in high‐populated, i.e., heavily urbanized areas of Tokyo and Kanagawa, Japan, focusing on landscape‐ and microhabitat factors simultaneously. Calling surveys were conducted at approximately 100 sites over 2 years (2022–2023) during the breeding season to identify key environmental variables influencing species occurrence. As a landscape factor, both species were positively associated with surrounding forest, suggesting the importance of landscape connectivity between forest as habitats and paddy fields as breeding sites. As a microhabitat factor, water retention in the paddies was a significant factor for both species. Notably, *R. schlegelii* depended on microhabitat features, in specific soil channels, whereas *D. japonica* did not. These findings suggest that while both species benefit from nearby forests in urbanized areas, only *R. schlegelii* is vulnerable to concreting that disrupts soil channels as breeding sites. The study underscores the need for conservation strategies that address both landscape‐ and microhabitat requirements to support amphibians in urbanizing agricultural regions. Importantly, species with similar ecological niches may respond differently to urban stressors, requiring species‐specific management strategies.

## Graphical abstract

Both frog species can persist in urbanized areas if forest habitats and paddy fields used for breeding remain. However, breeding site requirements differ between the species; concreting disproportionately impairs the reproductive success of *R. schlegelii* . While habitat loss is often emphasized as a key driver of amphibian decline, this study highlights the need to consider the entire life history when assessing threats.

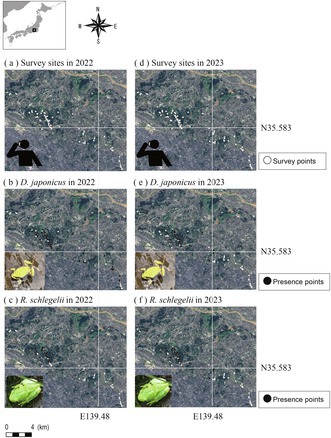

## 1 Introduction

Urbanization is rapidly increasing around the world (Seto et al. 2011, 2012). Unfortunately, it is one of the major anthropogenic alterations affecting terrestrial ecosystems and poses a significant threat to biodiversity (Beninde et al. 2015; Newbold et al. 2015; Fenoglio et al. 2020). Urbanized areas are complex and heterogeneous systems, characterized by fragmented, isolated, and degraded natural habitats (Grimm et al. 2008; Pickett et al. 2011). Therefore, understanding the effects of urbanization on diverse taxonomic groups is essential for improving biodiversity conservation practices (Dearborn and Kark 2010).

Amphibian populations have been declining globally in recent years (Stuart et al. 2004; Nyström et al. 2007; Arntzen et al. 2017; Luedtke et al. 2023). Urbanization severely impacts amphibian communities through habitat loss, fragmentation, isolation, and degradation, and currently threatens an estimated approximately 40% of amphibian species worldwide (Hamer and McDonnell 2008, https://www.iucnredlist.org/, accessed at 18, Nov, 2025). Amphibians require sufficient space and resources within aquatic and terrestrial habitats to fulfill critical life‐history processes, such as reproduction, foraging, and sheltering (Hamer and Parris 2011), and these habitats often need to be physically linked to allow dispersal between populations (Pope et al. 2000; Hamer and Parris 2011).

An overwhelming majority of empirical studies on the effects of urbanization on amphibians reviewed by Hamer and McDonnell (2008) report a decline in habitat availability and connectivity, resulting in lower occurrence, abundance, and species richness, as well as changes to community composition at relatively broad spatial scales. It is thus clear that urbanization negatively affects amphibian communities and may result in the local extinction of populations. However, previous studies have indicated that some amphibian species are relatively resilient to urbanization because they are habitat generalists or possess specific life‐history attributes (Hamer and McDonnell 2008; Hamer and Parris 2011).

The Japanese Tree Frog (*Dryophytes japonica*) and Schlegel's Green Tree Frog ( *Rhacophorus schlegelii* ) are both considered arboreal (Osawa and Katsuno 1997; Watabe et al. 2021; Matsushima and Hasegawa 2025). These two species exhibit similar ecological characteristics, including comparable body sizes and the use of rice paddies as breeding sites (Matsushima and Hasegawa 2025). Agricultural land including paddies often serves as an important habitat for amphibian species (Collins and Fahrig 2017; Li et al. 2020; Matsushima et al. 2022). However, *R. schlegelii* has been included in multiple regional Red Lists (Watabe et al. 2021), particularly in highly urbanized regions, including the Kanto and Kinki regions of Japan (https://ikilog.biodic.go.jp/Rdb/booklist, accessed at 18 Nov, 2025). Notably, *R. schlegelii* is listed in the Red Data categories in the three most populous prefectures in Japan, Tokyo, Kanagawa, and Osaka (https://www.stat.go.jp/data/jinsui/2024np/index.html#a05k01‐a, accessed at 18 Nov, 2025). The disparity in distribution patterns between these two species with similar ecological traits in urban areas suggests that *R. schlegelii* is more vulnerable to urbanization, while *D. japonica* may tolerate it to some degree.

A previous study demonstrated that habitat fragmentation caused by urbanization negatively impacts the distribution of both species (Matsushima et al. 2022). Additionally, *R. schlegelii* was rarely observed in areas with consolidated paddy fields, namely, those reorganized into large, consolidated plots with concretized irrigation channels and other modifications that facilitate mechanization, even in regions where the species was dominant (Osawa and Katsuno 1997). This has been attributed to the scarcity of soil embankments in consolidated paddies, which are crucial for egg‐laying, as they typically lay eggs in soil (Osawa and Katsuno 1997, 1999). In contrast, *D. japonica* is known to lay eggs in water with aquatic plants, submerged branches, and foliage (Seki and Matsui 2021); https://www.cgr.mlit.go.jp/ootagawa/Bio/amphi/index412.htm, accessed at 17 Nov 2025, suggesting that soil embankments do not constrain its reproductive capabilities. Based on these findings, it is considered that these two species can persist in urbanized landscapes, as long as both continuous forests (habitat) and paddies (breeding site) are present. However, it is hypothesized that while both species require a landscape structure with continuous forests (habitat) and rice paddies (breeding sites), each faces an additional limiting factor: *R. schlegelii* requires soil embankments as egg‐laying sites, and *D. japonica* requires water‐filled paddy fields. This difference is expected to influence the distribution patterns of the two species in urbanized regions. Although these individual factors have been identified in previous research, studies that simultaneously examine both factors across different spatial scales in urban landscapes are scarce.

Therefore, the objective of this study was to test the differences in distribution patterns between *D. japonica* and *R. schlegelii* in paddy fields in an urban landscape. We hypothesized that both frog species require a continuous landscape structure with forests and paddy fields as landscape factors, but each requires a specific microhabitat factor: *D. japonica* requires water‐filled paddies, and *R. schlegelii* requires soil embankments as egg‐laying sites. Based on the results, we discussed how to improve biodiversity in paddy fields for the conservation of wetland species in agricultural landscapes in urbanized regions.

## 2 Materials and Methods

### 2.1 Study Area

**FIGURE 1:**
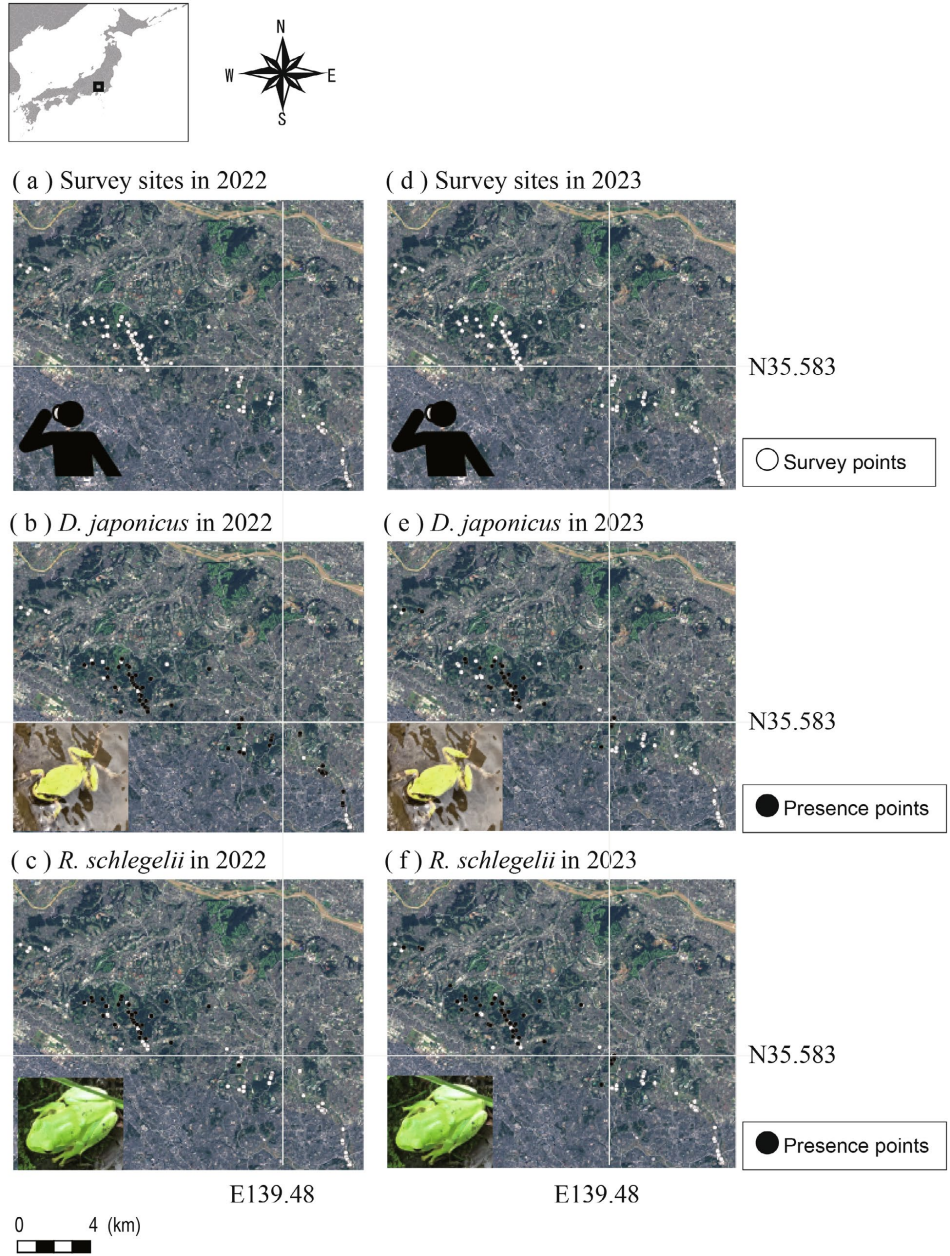
Survey sites in both 2022 (104 sites) and 2023 (94 sites) were basically the same, though the specific paddies surveyed sometimes differed. Aerial photographs were derived from Geospatial Information Authority of Japan (https://maps.gsi.go.jp, accessed 5 May 2025).

The survey was conducted in Machida City, Tokyo; and Yokohama City and Kawasaki City, Kanagawa, Japan (Figure 1). Machida City has a population density of 6023 persons per km^2^, while Yokohama and Kawasaki have densities of 8630 and 10,756 persons per km^2^ in 2020, respectively, among the higher levels in municipalities in Japan (https://www.e‐stat.go.jp/stat‐search/files?page=1&layout=datalist&toukei=00200521&tstat=000001049104&cycle=0&tclass1=000001049105&result_page=1&tclass2val=0, accessed at 18, Nov, 2025). Therefore, this region is considered largely urbanized, consisting of residential areas and green spaces, including broadleaf forests, paddy fields, dry farmlands, and orchard areas (Figure 1).

### 2.2 Study Species

The Japanese tree frog ( *D. japonica* ) is distributed throughout Japan (except the Ryukyu Islands), China, Korea, and Russia (Okuyama 2015; Seki and Matsui 2021). *D. japonica* is a semiarboreal forager inhabiting tall grasses and trees near paddy fields (Seki and Matsui 2021). It breeds in paddy fields after inundation in spring to summer (May to September) (Seki and Matsui 2021). Females appear at breeding sites only during spawning, while males remain in the paddy fields from May to September for advertisement calls (Seki and Matsui 2021). The Schlegel's Green Tree Frog ( *R. schlegelii* ) is distributed mainly around paddy fields in the Honshu, Shikoku, and Kyushu islands of Japan (Seki and Matsui 2021). This species uses paddy fields and irrigation ponds as breeding habitats, and paddy levees for foraging, escape, and overwintering (Osawa and Katsuno 1997, 1999). Breeding of *R. schlegelii* begins shortly after paddy field inundation in spring and continues into early summer (May to June) (Okuyama 2015).

### 2.3 Field Surveys

We conducted call surveys at 104 sites in 2022 and 102 sites in 2023 to determine the presence of *D. japonica* and *R. schlegelii* during their reproductive seasons, from May 9 to July 4 in 2022 and from May 8 to June 7 in 2023. The survey areas were kept as consistent as possible across the 2 years. However, in the second year, some sites could not be surveyed due to construction activities, restricted access, or development work in the paddy fields. Therefore, 10 surveyed paddies differed between 2022 and 2023. The surveyed paddies were basically the same, but points within the paddies differed in many cases.

During 5–10‐min nighttime surveys (from 19:00 to 24:00), we recorded the presence of calling individuals for the two species in the paddy fields. A previous study showed that a 5‐min survey was sufficient to confirm frog presence (Matsushima et al. 2022), but we extended the duration because frogs often stopped calling when approached. If frog presence was confirmed earlier, the survey was concluded even before 10 min had passed. Conversely, if the calling of target species was not heard during the 10‐min, it was recorded as absent.

Additionally, we conducted daytime field surveys to record microhabitat conditions. For each paddy field, we recorded the presence/absence of soil levees, concrete levees, soil channels, and concrete channels, as well as cultivation status (i.e., whether the paddy retained water). For initially dry paddies, additional visits were made during the survey term, and calling surveys were conducted if water had been added.

### 2.4 Landscape Structure Around the Study Site

Aerial photographs of the study area were used to generate polygon data for the sites using QGIS version 3.28 (https://www.qgis.org/, accessed 5 May 2025). We generated forest and paddy field polygon data covering all survey sites. The aerial photographs were from the Basic Map (Ortho Image, taken after 2007) provided by the Geospatial Information Authority of Japan (https://maps.gsi.go.jp, accessed 5 May 2025). Both land cover types could be clearly distinguished by visual inspection of the aerial photographs. We calculated the composition of the surrounding landscape for each site at two spatial scales—circular areas with 50 m and 100 m radii. These buffer distances were selected based on a previous study showing that forest and paddy area within 50 m significantly influence *D. japonica* habitat suitability (Tsuji et al. 2011). Additionally, most survey sites were within 150 m of one another, and a larger buffer would have caused excessive overlap.

### 2.5 Statistical Analysis

The effects of landscape and microhabitat factors on frog presence/absence were evaluated using a generalized linear mix effect model (GLMM) with binomial distributions and model selection based on the Akaike Information Criterion (AIC). The response variables were the presence/absence of *D. japonica* or *R. schlegelii* for each year. Prior to the statistical analyses, Moran's I statistics were calculated to assess whether the presence/absence patterns of the two frog species exhibited spatial autocorrelation. The results indicated significant spatial clustering for both species in both years (*p* < 0.001). Therefore, a spatial random effect based on the Matern covariance function was incorporated into the statistical models (Lindgren et al. 2011). Also, to ensure sufficient variability, the data from the 2 years were pooled, and “year” was included as a random effect.

A two‐step modeling approach was applied. First, the best model for landscape features was identified based on forest and paddy areas within 50 or 100 m buffers. Eight variable combinations were tested: models using a single variable (one for forest or paddy) and models using paired variables (two combinations per land cover type). The model with the lowest AIC value was selected. Note that forest and paddy variables from both 50 m and 100 m buffers were never included simultaneously in the same model.

Second, the best model for microhabitat features was selected based on the presence/absence of concrete levees, soil channels, and cultivation status. Soil levees and concrete channels were excluded, as over half the survey sites had these features. Although nearly all 2022 sites had water retained, this was included as an explanatory variable due to its relevance to the hypothesis. In 2023, about half the sites lacked retained water. As with the landscape model, the best combination of explanatory variables was chosen based on the lowest AIC value. Finally, variables from the best landscape and microhabitat models were combined to construct the final models. Of the final models, we tested the explanatory variables for multicollinearity by calculating variance inflation factors (VIFs) (VIF < 2 for both final models).

All statistical analyses were conducted using R version 4.5.0 (R Development Core Team). The “fitme” command from the “spam” package was used to determine the GLMM model with spatial random effect. The “fitme” command from the “car.” Package was used to determine the VIF values.

## 3 Results

In 2022, we detected 71 and 35 occurrences of *D. japonica* and *R. schlegelii* at 104 survey sites (Figure 1a–c, Table 1). In 2023, 44 and 46 occurrences for *D. japonica* and *R. schlegelii* at 102 survey sites (Figure 1d–f, Table 1). In both 2022 and 2023, almost all survey sites had soil levees, and over half had concrete channels (Table 1). In 2022, almost all survey sites have water retain but in 2023, about half of the sites did not have water retain (Table 1). The paddy areas within the 50‐m buffer were 3283.6 ± 2059.9 m^2^ (average ± SD) and within the 100‐m buffer 6444.8 ± 4676.5 m^2^, respectively. The forest areas within the 50‐m buffer were 1054.99 ± 1423.6, and within the 100‐m buffer 7638.95 ± 7555.1 m^2^, respectively.

**TABLE 1:** Results of the field survey in both 2022 and 2023. The number of 50‐m survey units where each survey item was confirmed is indicated (figures in parentheses show the percentage relative to the total units of each course).

Year	Number of survey points	Number of occurrences o*n Hyla japonica*	Number of occurrences on *Rhacophorus schlegelii*	Number of occurrences on soil levees	Number of occurrences on concrete levees	Number of occurrences on soil channel	Number of occurrences on concrete channel	Cultivation status (1: water retain, 0: not water retain)
2022	104	71	35	101	28	47	53	94
2023	102	44	46	92	38	30	68	49

In the landscape model of *D. japonica* , forest areas within the 50 m buffer were positively correlated with the occurrences (Table 2). Of *R. schlegelii* , forest areas within the 100 m buffer were positively correlated with the occurrences (Table 2).

**TABLE 2:** GLM and model selection results for the occurrences of *Dryophytes japonica* and *Rhacophorus schlegelii* using landscape variables.

Explanatory variables	Presence/absence of *Dryophytes japonica*	Presence/absence of *Rhacophorus schlegelii*
Paddy areas within 50 m buffer	—	—
Paddy areas within 100 m buffer	—	—
Forest areas within 50 m buffer	1.07e^−4^ ± 1.52e^−4^	—
Forest areas within 100 m buffer	—	9.99e^−5^ ± 4.24e^−5^
Intercept	0.6 ± 0.3	−3.30 ± 1.98
AIC	251.31	191.78

*Note:* Values indicate estimated coefficients ± S.E. “—” indicates variables not included in the lowest AIC models.

In the microhabitat model of *D. japonica* , the cultivation status, namely the presence/absence of water retained in the paddy fields, was positively correlated with the occurrences (Table 3). Of *R. schlegelii* , soil channels and cultivation status were positively correlated with the occurrences in 2023 (Table 3).

**TABLE 3:** GLM and model selection results for the occurrence of *Dryophytes japonica* and *Rhacophorus schlegelii* using microenvironmental variables.

Explanatory variables	Presence/absence of *Dryophytes japonica*	Presence/absence of *Rhacophorus schlegelii*
Concrete levees	—	—
Soil channel	—	0.90 ± 0.52
Cultivation status	4.93 ± 0.77	2.39 ± 0.68
Intercept	−0.98 ± 0.7	−4.16 ± 2.099
AIC	175.095	180.27

*Note:* Values indicate estimated coefficients ± S.E. “—” indicates variables not included in the models.

Based on the results of both landscape‐ and microhabitat model analysis, the final models are shown in Table 4. The AIC values for *D. japonica* model were lower than that of the landscape model but a little higher than that of the microhabitat model (Table 4). On the other hand, *R. schlegelii* models were lower than those for the landscape‐ and microhabitat models (Table 4).

**TABLE 4:** GLM and model selection results for the occurrence of *Dryophytes japonica* and *Rhacophorus schlegelii* using both landscape and microenvironmental variables.

Explanatory variables	Presence/absence of *Dryophytes japonica*	Presence/absence of *Rhacophorus schlegelii*
Paddy areas within 50 m buffer	—	—
Paddy areas within 100 m buffer	—	—
Forest areas within 50 m buffer	1.02e^−4^ ± 1.47e^−4^	—
Forest areas within 100 m buffer	—	9.87e^−5^ ± 3.33e^−5^
Concrete levees	—	—
Soil channel	—	6.22e^−1^ ± 4.47e^−1^
Cultivation status	4.97 ± 0.78	2.74 ± 6.395e^−1^
Intercept	3.98 ± 0.89	−5.21 ± 2.24
AIC	176.65	173.15

*Note:* Values indicate estimated coefficients ± S.E. “—” indicates variables not included in the models.

## 4 Discussion

This study tested the differences in the distribution patterns of two frog species inhabiting paddy fields in urbanized areas, considering both landscape and microhabitat factors. Results showed that both species require the forest, but the breeding‐related microhabitat factors differed. *D. japonica* appeared to require water retention only, whereas *R. schlegelii* requires both water retention and soil channel. Our hypothesis was basically supported. This difference is believed to underlie the situation in which only *R. schlegelii* faces extinction risk.

In both 2022 and 2023, field surveys were conducted across Machida, Yokohama, and Kawasaki Cities, and few paddy field areas lacked detectable frog calls. *D. japonica* and *R. schlegelii* —especially *D. japonica* —were dominant and present at many sites. Studies from around 2000 also reported these species as dominant in nearby rice paddies (Osawa and Katsuno 1997), suggesting stable populations over time.

Crucial landscape factors were generally similar for both species. For distribution of *D. japonica* was positively associated with forest area within a 50‐m buffer. *D. japonica* is a forest‐dwelling species that uses rice paddies mainly for breeding (Watabe et al. 2021). Thus, large paddy field areas may not be essential; rather, paddies connected to surrounding forests are likely more important (Tsuji et al. 2011). This supports the result clearly. For *R. schlegelii* , forest area within a 100‐m buffer showed a positive association. Like *D. japonica* , *R. schlegelii* is a forest‐dwelling species that breeds in paddy fields (Osawa et al. 2001; Watabe et al. 2021). Thus, the continuity between forests and paddies is essential. These findings suggest that forest presence surrounding paddy fields is a key landscape factor for both species, linking habitat and breeding sites in urbanized areas. Actually, previous studies have also indicated that forests surrounding agricultural fields are strongly associated with amphibian diversity (Collins and Fahrig 2017).

Microhabitat factors were basically the same for both species, but key factors differed. For *D. japonica* , water presence in paddies was positively associated with occurrence, consistent with a previous study (Naito et al. 2012). For *R. schlegelii* , both water retained and soil channels were positively related to occurrence. *D. japonica* lays eggs on aquatic vegetation and substrates (Seki and Matsui 2021); https://www.cgr.mlit.go.jp/ootagawa/Bio/amphi/index412.htm, accessed at 5 May 2025, making water retained essential. Prior studies emphasized in‐paddy conditions for *D. japonica* breeding (Naito et al. 2012), supporting this result. In contrast, *R. schlegelii* lays eggs in soil (Osawa and Katsuno 1997; Osawa et al. 2001) and cannot breed without exposed soil. Soil channels are important as oviposition sites and for larval movement into water. Despite having versatile toe pads, *R. schlegelii* may struggle to climb artificial structures like U‐shaped concrete channels (Watabe et al. 2013), which may have influenced results. Therefore, *R. schlegelii* is more vulnerable to urbanization. The water retained may reflect impacts of field abandonment or fallowing, where lack of irrigation dries nearby channels, limiting larval access to water and discouraging oviposition. These findings suggest that, at the microhabitat scale, suitable oviposition sites are limiting for both species.

While this study yielded intriguing results regarding differences in the distribution patterns of *D. japonica* and *R. schlegelii* , some limitations in the survey design existed. First, although the survey spanned 2 years, each year included only a single survey event, which represents a methodological limitation. For example, Matsushima et al. (2022) conducted two surveys within one season, and the results of this study might have differed if multiple surveys had been conducted. Specifically, the 2023 survey period was about 1 month shorter than in 2022, and the number of paddies without standing water increased sharply. This was due not only to more fallow paddies but also to the irrigation schedule that year. If an additional survey had been conducted after those initially dry paddies were later flooded, detection patterns of the focal species might have changed. Another limitation was that although the same sites were targeted in both years, specific survey points sometimes differed, or their conditions changed due to fallowing or other factors. This was primarily because rice paddies are privately owned, making it infeasible to mark locations directly and difficult to manage agricultural activity. Additionally, access was restricted at some sites during the 2023 agricultural season. Despite this, presence data were collected at the level of individual paddies for each survey, likely minimizing the impact of this limitation on the overall results.

## 5 Conclusions

This study clearly demonstrated differences in urbanization tolerance between *D. japonica* and *R. schlegelii* , two frog species with similar ecological traits. For both, continuity between forest habitats and breeding‐use paddy fields was important. However, a marked difference existed in their tolerance to breeding site modifications. *D. japonica* appeared tolerant of agricultural infrastructure modernization, including concreting, whereas *R. schlegelii* was presumed unable to reproduce in concreted areas. Forest loss leads to habitat disappearance, which could drive both species toward extinction—a relatively straightforward process, although *D. japonica* might have been tolerant at some degree. In contrast, concreting of paddy infrastructure, often accompanying urbanization and modernization, may pose a subtler but significant threat affecting only *R. schlegelii* . For instance, even if irrigation channels are concreted, this may not immediately affect *R. schlegelii* survival. However, inhibited reproduction under such conditions may cause gradual species decline over the medium term. Various urbanization effects on amphibians have been identified, but these are often interrelated. Even species tolerant to one stressor may face extinction if vulnerable to another. To conserve amphibian habitats in urban areas, conservation strategies must address multiple factors affecting life histories of the species, ensuring none of these essential elements are compromised.

## Author Contributions

**Takeshi Osawa:** conceptualization (equal), data curation (equal), formal analysis (equal), funding acquisition (equal), investigation (supporting), project administration (lead), writing – original draft (lead), writing – review and editing (lead). **Nozomu Sato:** investigation (equal), methodology (equal), writing – review and editing (supporting). **Hiroto Nagaoka:** investigation (equal), writing – original draft (equal).

## Conflicts of Interest

The authors declare no conflicts of interest.

## Supporting information


**Data S1:** code2year.

**Data S2:** data2year.

## Data Availability

Analyzed data sets and analyzed code were provided as Supporting Information. All data sets could be provided based on reasonable request.
